# Effects of Speciation, Cooking and Changes in Bioaccessibility on Methylmercury Exposure Assessment for Contrasting Diets of Fish and Marine Mammals

**DOI:** 10.3390/ijerph18052565

**Published:** 2021-03-04

**Authors:** Tania Charette, Gregory Kaminski, Maikel Rosabal, Marc Amyot

**Affiliations:** 1 Groupe de Recherche Interuniversitaire en Limnologie (GRIL), Université de Montréal, Département de Sciences Biologiques, Complexe des Sciences, C.P. 6128, succ. Centre-Ville, Montréal, QC H3C 3J7, Canada; tania.charette@umontreal.ca; 2 Health Canada, 269 Laurier West, Ottawa, ON K1A 0K9, Canada; greg.kaminski@canada.ca; 3 Groupe de Recherche Interuniversitaire en Limnologie (GRIL), Département des Sciences Biologiques, Université du Québec à Montréal (UQAM), 141 Avenue du Président-Kennedy, Montreal, QC H2X 1Y4, Canada; rosabal.maikel@uqam.ca

**Keywords:** methylmercury, exposure, fish, mammal, probabilistic

## Abstract

Uptake of the neurotoxicant monomethylmercury (MeHg) from fish and marine mammals continues to present a public health concern in Canada and elsewhere. However, fish and marine mammals are key diet items contributing to food security for some Indigenous populations in Canada. Mercury (Hg) exposure is estimated assuming that 100% of Hg is methylated, that 100% will be absorbed by the consumer and that cooking does not affect MeHg concentrations. Some of these assumptions do not correspond to our current state of knowledge. The aim of this study was to assess the impact of additional variables on Hg exposure equation using probabilistic risk analysis. New variables tested were (1) the proportion of methylated Hg compared to total Hg (pMeHg, %), (2) the relative absorption factor (RAF, %) expressed as bioaccessibility and (3) the mass loss factor (MLF, unitless) that represents the loss of moisture during cooking, known to increase MeHg concentration in fish and mammals. For the new variables, data from literature were used in order to set point estimate values that were further used in the probabilistic risk analysis. Modelling results for both fish and marine mammals indicate that adding these new variables significantly influenced estimates of MeHg exposure (Mood’s median test, *p* < 0.05). This study highlights that the evaluation of exposure to MeHg is sensitive to pMeHg, RAF and MLF, and the inclusion of these variables in risk assessment should be considered with care. Further research is needed to provide better food-dependent, population-specific estimates of RAF and MLF before formal inclusion in exposure estimates.

## 1. Introduction

Exposure to monomethylmercury (MeHg) from fish and marine mammals continues to present public health concerns in Canada and elsewhere [1,2]. This Hg chemical species is known for its ability to cross mammal blood–brain and placental barriers [3,4,5]. Studies have shown harmful neurological and neurodevelopmental effects due to MeHg exposure in babies and young children linked to maternal consumption of fish with elevated MeHg levels during pregnancy [6,7]. Populations consuming marine mammals are at greater risk, such as the Inuit population from Canada, since marine mammal meat and offal may contain high levels of MeHg and are often consumed [2]. A mean concentration of blood total Hg (THg) level of pregnant women from Nunavik was established at 4.2 μg/L (data from 2017) [8], compared to 0.6 μg/L for pregnant women from the general Canadian population (data from 2008 to 2011) [9].

Country food can be defined as « all food within a particular culture available from local natural resources and culturally accepted » [10]. Its consumption may present toxicological risks but remains desirable for several reasons. Country food consumption contributes to physical, mental, spiritual, emotional and social health in Indigenous populations [11]. Country foods are rich in proteins, vitamins, minerals and poor in saturated fats and sugars [12], which are key factors in preventing chronic diseases [11]. Furthermore, access to country food contributes to food security and helps to overcome lack of money for vulnerable households [13], since commercial food in the Canadian North is much more expensive than in the South [14].

In Canada, the consumption guidelines use a tolerable daily intake (TDI) as a basis for the calculation of allowable portion size and frequency of consumption [15]. Tolerable daily intake represents the reference dose (μg × kg^−1^ body weight (bw) per day) for a non-carcinogenic compound that can be consumed daily over a lifetime without any harmful effects on human health [16]. This is based on the notion that harmful effects begin beyond a certain threshold [17]. Methylmercury TDIs determined by Health Canada are set at 0.2 μg × kg^−1^ bw per day for sensitive populations (women of childbearing age and children < 12 years) and 0.47 μg × kg^−1^ bw per day for the general adult population [15]. It is not identical worldwide: WHO/FAO sets a benchmark dose of 0.23 μg × kg^−1^ bw per day [18], whereas U.S. EPA determined the threshold to be 0.1 μg × kg^−1^ bw per day (including sensitive subgroup) [19].

The equations used by Health Canada [15] in the calculation of Hg exposure defined by the current model (*Ecm*) and hazard quotient (*HQcm*) are:

(1) $$Ecm\;=\frac{CR\times \left[THg\right]}{BW}$$

(2) $$HQcm=\frac{Ecm\;\left(\mathrm{mg}/\mathrm{kg}\;\mathrm{bw}\;\mathrm{per}\;\mathrm{day}\right)}{pTDI\;\left(\mathrm{mg}/\mathrm{kg}\;\mathrm{bw}\;\mathrm{per}\;\mathrm{day}\right)}$$

where *CR* represents the consumption rate (mg wet weight (ww) fish flesh per day), *[THg]* is the THg concentration in the fish flesh (mg × kg^−1^ ww) and *BW* is for body weight (kg). Hazard quotient is used for threshold chemical compounds (non-carcinogenic), such as MeHg [16] and links the exposure and the potential risk [20]. HQ ≤ 1 should not cause health effects, whereas HQ > 1 could imply risks for exposed individuals. The assessment of MeHg exposure through food using Equation (1) implies that 100% of Hg is present as MeHg. However, while the proportion of MeHg compared to THg is high in fish species (>80%) [21], it is lower in marine mammal meat (range from 65 to 86%) and offal (range from 8 to 26%) [2]. Moreover, Equation (1) assumes that 100% of MeHg will be absorbed by the consumer, yet this assumption is based on a 1970s study, where humans were exposed to a solution of MeHg nitrate [22]. Furthermore, a growing number of in vitro digestion studies suggest that fish flesh cooking could decrease MeHg solubilization in digestive fluids (i.e., bioaccessibility), theoretically leading to a lower absorption rate [23,24,25,26,27].

At the moment Health Canada recommends a deterministic approach to assess MeHg exposure (see Equation (1)) through consumption pathways [15], involving the use of a point estimate value to describe each variable of Equation (1) neglecting the potential variability within population and uncertainties in the data [28]. In comparison, probabilistic analysis uses probability distributions defining one or more variables, accounting for the variability and uncertainties and resulting in a full range and frequency of hypothetical risks [28]. Often, upper-bound (worst-case scenario, 95th percentile) estimate variables are used in a deterministic approach to compensate for not taking the range of variability and data uncertainties into consideration. This also increases the confidence that risks have not been underestimated [15,29].

This paper aims to assess the impact of the inclusion of new variables in the MeHg exposure equation using probabilistic analysis. This analysis was performed for two consumption scenarios: Scenario 1 is based on the consumption patterns of the general Canadian population (consuming mostly salmon and albacore canned tuna) while scenario 2 is based on consumption practices specific to many northern Indigenous populations (seal liver, beluga meat and beluga *nikku*). For scenario 1, we assessed MeHg exposure of the general population and of the sensitive part of the population, for medium (22 g/day) and high (40 g/day) consumption rates of fish. In scenario 2, we assessed the exposure to MeHg for the general Indigenous population and the sensitive part of this population, using consumption rates specific to each mammal species assessed.

We only considered fish and mammal meats as MeHg exposure pathways since other MeHg sources are considered negligible [30]. Furthermore, only MeHg was used in this risk assessment but it is known that fish and mammal muscles can contain other contaminants [31,32]. Thus, this study does not constitute a full human health risk assessment and was intended to be more of a sensitivity analysis. The goal of this paper is to test the exposure calculation by taking into account recent research. We consider that our study will serve as a starting point for practitioners and scientists to adjust currently used exposure equations, taking into consideration specific consumer groups and their diet. We do not propose any health-related changes in consumption guidelines, since we simply explore the potential effect of introducing new terms in exposure assessments, in order to guide future research.

## 2. Materials and Methods

### 2.1. Population and Diet

Our study compared MeHg exposure for two different dietary intake scenarios. Scenario 1 (Table 1) reflects the general Canadian diet and considers MeHg intake from salmon and canned tuna. Salmon (all species combined) was selected because it is the fish most consumed in Canada [15]. We also chose Albacore canned tuna containing generally relatively high Hg levels, and for which Health Canada issued consumption guidelines, in contrast to canned light tuna containing less Hg [33]. For the general and sensitive population, CR values and THg levels for salmon and canned tuna were obtained from Health Canada’s publication [15], and BW from Canadian Exposure Factors Handbook [34] (Table 1). Scenario 2 (Table 1) reflects dietary consumption by the Indigenous Canadian population for which BW, CR and mass loss factor (MLF) data were obtained from Lemire et al. [2]. Lemire et al. based their data on the 2004 Nunavik Health Survey; we reevaluated the data on MeHg exposure for key marine mammal species using a probabilistic approach. The Indigenous BW value is a geometric mean (*n* = 702, where women represent 48.2%) adjusted for age and gender, which could have overestimated the BW of the sensitive population in our modelling. THg concentration in marine mammals was obtained from Palaniyandi [35]. Beluga *nikku* (air-dried beluga meat) and seal liver were chosen to represent the marine mammal diet because they are the most Hg-contaminated part of the northern country diet [2,35,36]. In order to assess the effect of drying (MLF) on THg levels, beluga meat THg value was used in the modelling of risk assessment (Table 1). Note that scenario 2 is not meant to represent any specific community but rather aims to consider a diet rich in marine mammals. No inference to risk assessments of specific communities or populations should be derived from this theoretical exercise. The variables used in each scenario are described in detail in Table S1.

### 2.2. New Proposed Variables

We tested an alternative model to the current *Ecm* model represented by Equations (1) and (2) using the following equation:

(3) $$Eam=\frac{CR\times \;\left[THg\right]\;\times \;pMeHg\;\times \;RAF\times \;MLF}{BW}$$

where *Eam* is the exposure estimate with the alternative model, *pMeHg* corresponds to the proportion (%) of THg that is methylated in the tissue, *RAF* relates to the relative absorption factor (RAF) (expressed as bioaccessibility, %) and MLF means mass loss factor (MLF, unitless) in order to compensate for the moisture loss during cooking. Point estimates (central tendency exposure; CTE) were used in the modelling using Equation (3) (Table 1). For pMeHg in fish flesh, two values were used: 100% (as the value currently used by health authorities [15]) and 90% as a value presented in the general literature (according to the European Food Safety Authority that reports 26 studies describing a mean range between 80% and 100%) [30]. Values for beluga meat and seal liver were age-adjusted (16.5 and 6 years, respectively) based on a study by Lemire et al. [2]. For RAF values, we chose 100% (value used by default by health authorities, as they consider a complete absorption of MeHg) [15] and 40% as a mean MeHg bioaccessibility reported in studies of cooked fish using different species, such as swordfish, grouper, tuna, salmon, common smooth-hound, Atlantic wreckfish, black scabbardfish, shark, tilapia, snapper, turbot and anchovy [24,25,37,38]. We allocated an RAF of 40% to canned tuna since it is cooked before being canned [45]. In the literature, the data on RAF for marine mammals are very scarce and no study was found describing the bioaccessibility of MeHg from this country’s food. However, some studies have reported bioaccessibility of THg; an RAF of 51% was reported for beluga meat [35], and an average of 33% for beluga *nikku* [35,39]. An average of 27% was associated with ringed seal liver [35,39,40]. In this study, bioaccessibility values were used since no in vivo study assessed the relative absorption of MeHg from cooked flesh. Finally, we included MLF in Equation (3) when appropriate in order to represent the loss of humidity during cooking, which is known to increase the MeHg concentration in fish flesh [37,41,46,47,48]. The value of 1.35 encompasses all cooking methods (grilling, frying, boiling, etc.) [37,41,46,47,48]. No MLF factor was added in the case of canned tuna that does not need further food processing before being eaten, and seal liver that is consumed raw [2]. The MLF for beluga *nikku* corresponds to the loss of moisture during air-drying of the beluga meat and was set at 2.5 [2].

The following equation was used to calculate the CR of canned tuna that would be allowed if HQ equals 1:

(4) $$CR\;limit=\frac{pTDI*BW*HQ}{\left[THg\right]*pMeHg*RAF}$$

### 2.3. Probabilistic Risk Assessment

For this study, CTE characterizes the mean whereas reasonable maximum exposure (RMaE) represents the 95th percentile of CTE (Table 1). Point estimate (CTE) values were used for the new parameters of Equation (3) (RAF, pMeHg and MLF) (Table 1). On the other hand, in order to account for the potential variability in the population (Table 1, BW) and uncertainties in the data (Table 1, [THg]), we developed a probabilistic risk assessment according to Health Canada [28] and U.S. EPA [29] procedures with R software [49], using a first-order Monte Carlo one-dimensional simulation (*n* = 10,000). Typically, 10,000 iterations are appropriate to capture most of the variability of the input distributions extremities [28]. The stability of the results was tested by varying the number of iterations from 10,000 to 10,000,000 and similar results were obtained (Table S2). We generated lognormal distributions (*n* = 10,000, R: *rlnormTrunc* function from *EnvStats* package) for [THg] and BW with defined values based on literature (Table 1). According to Health Canada [28] and U.S. EPA [29], variables subject to the multiplicative effect of a large number of processes tend to yield a lognormal shape distribution. For Monte Carlo analysis, dependencies between variables were considered negligible. The only potential dependency that may occur is a positive correlation between the CR and the BW [15], but since they are positioned at both sides of the exposure equation (CR at the numerator and BW at the denominator, see Equations (2) and (3)) this limits the bias caused by this dependency.

### 2.4. Impact of RAF on Risk Estimates

To illustrate the impact of MeHg bioaccessibility on the level of estimated risk (expressed as HQ; we extracted data from various studies [37,50,51,52,53,54,55]. We used the values of MeHg bioaccessibility and MeHg initial concentration in fish muscle in order to calculate the resulting HQ. Equation (1) (CR-medium consumption and BW of the general population, Table 1) with the addition of RAF was applied in the modelling.

### 2.5. Statistic and Icons

The resulting HQ distributions were summarized with box-and-whisker plots. When needed, HQ distributions were compared with Mood’s median test. Significance level was set at α < 0.05. Seal and beluga icons were downloaded from the noun project (www.thenounproject.com; accessed on 5 January 2021), from Victoruler, Valeriia Vlasovtseva artists, ProSymbols and Rfourtytwo.

## 3. Results

### 3.1. The Impact of the Proposed Variables on Methylmercury Exposure

Table 2 summarizes the MeHg daily exposure dose as a function of diet, CR and BW for each scenario, and the impact of adding a single variable in a stepwise way to the exposure equation. Only albacore canned tuna results are presented since salmon did not produce exposure superior to TDI. The use of the current model (Equation (1)) for the Canadian non-Indigenous population, led to estimates of daily doses below the TDI of 0.47 μg × kg^−1^ bw per day for the general adult population. The addition of each single variable led to an exposure distribution significantly different from *Ecm*.

The TDI for sensitive population (women of childbearing age and children < 12 years) is 0.2 μg × kg^−1^ bw per day and this value was exceeded by 4% and 50% of the population regarding the canned tuna scenario for the medium and high consumption rate respectively, when using the current model (see Equation (1)). In the high consumption rate scenario, the addition of pMeHg and RAF decreased the percentage of the population exceeding the TDI by 12% and 50%, respectively. For this scenario, the use of the alternative model led to a safe MeHg exposure in 99.9% of cases. For the seal liver scenario, the addition of either pMeHg or RAF decreased the percentage of at-risk populations to zero %, in comparison to the TDI obtained with the current model (see Equation (1)) which led to 2.7% of the at-risk population. For the beluga *nikku*, 0.1% of the population exceeds the TDI when MLF is considered. Overall, while pMeHg and RAF lead to a decrease in exposure, MLF increases it. These results suggest that the proposed variables (pMeHg, RAF and MLF) should be considered in exposure modelling by health authorities.

### 3.2. Risk Characterization

**Figure 1:**
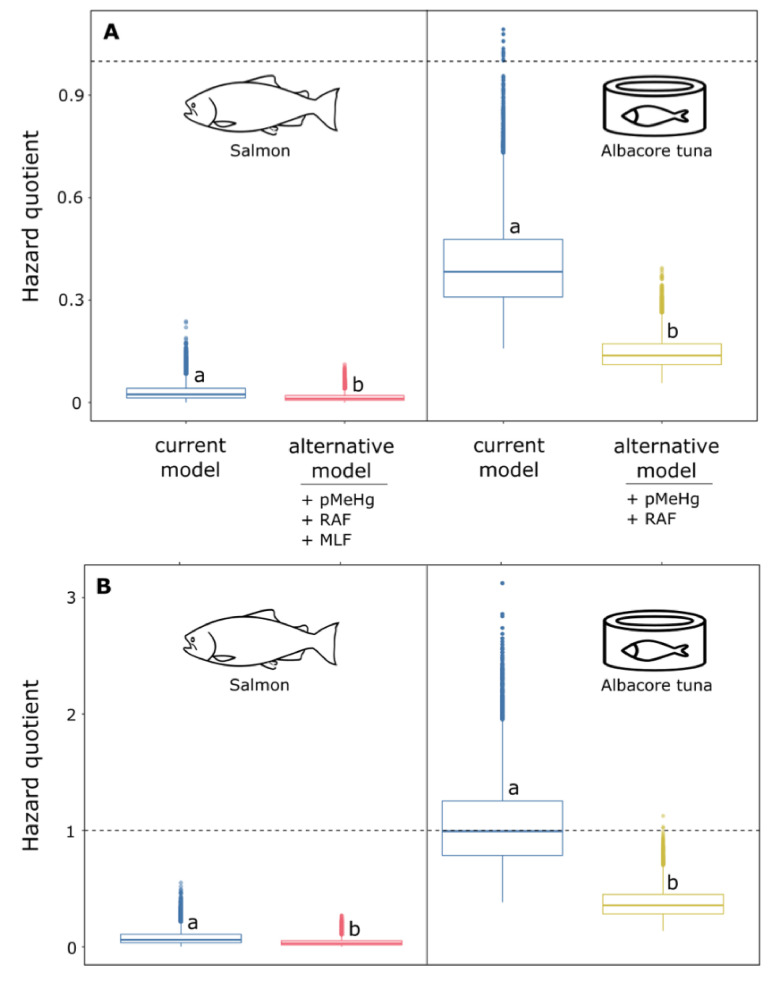
Hazard quotient as function of the fish species consumed and as function of the variables used in the assessment of MeHg exposure. High consumption rate scenario was used. (**A**) Canadian general population and (**B**) sensitive Canadian general population. Letters are used to indicate a significative difference (*p* < 0.05) between HQ distribution of a given fish species. See Table S1 for the details of variables used for each scenario. pMeHg: proportion of THg that is methylated; RAF: relative absorption factor; MLF: mass loss factor.

**Figure 2:**
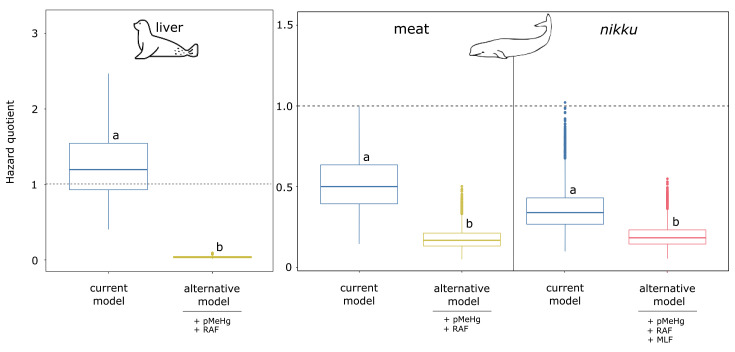
Hazard quotient as function of marine mammals consumed and as function of the variables used in the assessment of MeHg exposure for the sensitive Indigenous population. Letters are used to indicate a significative difference (*p* < 0.05) between a given scenario. See Table S1 for the details of variables used for each scenario. pMeHg: proportion of THg that is methylated; RAF: relative absorption factor; MLF: mass loss factor.

In order to assess the impact of the proposed variables on the health risk from MeHg exposure, we compared the HQ distributions obtained using *Ecm* vs. *Eam*. As observed in Figure 1 and Figure 2, the simultaneous addition of all proposed variables (pMeHg, RAF and MLF) systematically led to a decrease of MeHg risk exposure.

#### 3.2.1. Salmon and Canned Tuna Consumption Scenario

Figure 1 shows the HQ distribution as a function of the fish species consumed frequently and as a function of the variables used in the assessment of MeHg exposure, for the general and sensitive Canadian population. According to Figure 1, salmon consumption should not cause any health risk; using the current model we estimated HQ (median) to be between 0.01 and 0.03 for the general and the sensitive population, respectively, (Table S3). On the other hand, the consumption of canned tuna by the sensitive Canadian general population using the current model (see Equation (1)) led to HQ > 1 for 50% of the population (Table S3), whereas the addition of pMeHg and RAF into the assessment produced an HQ distribution where 99.9% of the population was not exposed to any risks (HQ < 1).

#### 3.2.2. Marine Mammal Consumption Scenario

Figure 2 illustrates the health risks associated with MeHg exposure through marine mammal consumption with the example of the sensitive Indigenous population. In the case of the seal liver, which is consumed raw, the current model (see Equation (1)) led to HQ > 1 in 69% of cases, whereas the addition of pMeHg produced an HQ above 1 in 100% of cases (Table S3). Regarding the beluga meat and *nikku*, the use of the current model (see Equation (1)) produced HQ > 1 for 1.9% and 0.01% of the population, respectively).

### 3.3. The Implication of RAF in Methylmercury Risk Characterization

**Figure 3:**
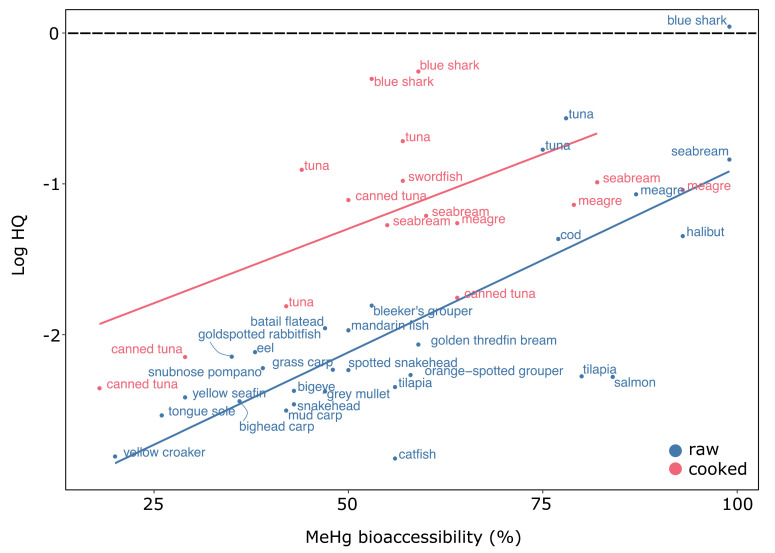
Impact of RAF (expressed as bioaccessibility, %) on HQ. Blue for the raw bioaccessibility value and pink for the cooked one. Dotted line represents HQ = 1. HQ was assessed using the CR-medium consumption and BW of the general population (Table 1). The initial MeHg level in fish flesh and their respective bioaccessibility were obtained from: [37,50,51,52,53,54,55].

We explored the relationship between RAF (expressed as bioaccessibility, %) and HQ, to assess the effect of cooking on HQ, since cooking has been shown to decrease the MeHg bioaccessibility in in vitro studies [23,24,25,26]. Figure 3 was created using the values (*n* = 45) of seven studies that have assessed the effect of cooking on MeHg bioaccessibility in the flesh of various fish [37,50,51,52,53,54,55]. Methylmercury exposure used to estimate HQ was calculated using the CR-medium consumption and BW of the general population (Table 1), the initial MeHg level in fish flesh and their respective bioaccessibility. Hence, we used the current model + RAF. As mathematically expected, HQ is linearly related to the bioaccessibility (raw: *r*^2^ = 0.50, *p* < 0.05; cooked: *r*^2^ = 0.35, *p* < 0.05). According to Figure 3, the risk related to the consumption of cooked fish is higher than for raw fish, which is associated with higher MeHg levels in species chosen for the assessment of the effect of cooking on bioaccessibility. It is noteworthy that for a given fish species, bioaccessibility may largely vary.

## 4. Discussion

### 4.1. The Proposed Model Modifications Impact Methylmercury Exposure Assessment

In all cases, every suggested variable significantly impacted MeHg daily dose exposure compared to the current model (see Equation (1)) (Table 2). For instance, according to Health Canada guidelines, sensitive members of the population should limit their consumption of albacore canned tuna to 300 g per week [33], which is seven times less than the quantity that could be allowed when the proposed variables are included in the assessment of MeHg exposure (see Equation (4), data not shown). This study, therefore, highlights the importance of carefully considering the proposed variables in MeHg exposure assessment and the need to better estimate these variables with care.

The value of 90% used for the pMeHg (Table 1) for fish flesh is still quite conservative since pMeHg tends to vary according to the trophic level, feeding habits and age. Lescord et al. [56] found a pMeHg range from 40 to 100% in Canadian fish flesh, and this is in agreement with the conclusion of the Evaluations of the Joint FAO/WHO Expert Committee on Food Additives (JECFA) who provided a range between 30% and 100% [7]. The value of pMeHg for marine mammals varies in literature as well. For instance, values for beluga muscle pMeHg range from 65 to 97% [2,57,58]. Our study may underestimate MeHg exposure since we chose the inferior value of this range (Table 1). In seal liver, literature shows that pMeHg can be as low as 2.7% and is known to decrease with the age of the seal [58], complexifying the assessment of MeHg exposure.

Another variable that we suggest adding in the assessment of MeHg exposure is RAF, which decreased significantly the exposure of MeHg (Table 2, Figure 3). Bioaccessibility studies have demonstrated that MeHg solubility decreased with fish flesh cooking [24,25,37,38]. However, it is important to note that bioaccessibility values are notoriously variable with low inter-laboratory or even intra-laboratory reproducibility, and the lack of use of standardized protocol leads to a wide range of values [25]. Additionally, the impact of cooking on MeHg oral bioavailability was assessed using pigs and it was suggested that cooking fish muscle did not modify MeHg oral bioavailability [59]. Hence, currently, in vitro and in vivo results diverge.

A standardized in vitro digestion protocol was developed by the COST INFOGEST network, in which skim milk powder protein digestibility was validated using pig models [60]. Once optimized for metals, this model protocol could be used as a starting point to standardize in vitro protocols for MeHg bioaccessibility studies, since MeHg is mostly bound to proteins. It could also be used for the assessment and validation of in vitro digestibility of raw and cooked fish flesh.

Presently, we do not recommend using bioaccessibility (i.e., RAF) estimates in the assessment of MeHg exposure, since it could significantly underestimate the exposure of the consumer without sufficiently strong scientific evidence. Additionally, since pMeHg highly varies in country foods [2], THg bioaccessibility could be a weak proxy of bioaccessibility of MeHg. Standardization and in vivo validation are needed before RAF is included in the alternative model.

The last variable studied was MLF. While there is no consensus regarding the impact of cooking on MeHg levels, the majority of studies point in the direction of an increase of MeHg concentration due to mass loss linked to water loss during cooking [37,41,46,47,48]. Including MLF in the model increases drastically the daily exposure to MeHg, but on the other hand, according to bioaccessibility literature, cooking [24,25,37,38] and drying [35,39] can decrease the solubilization of MeHg and THg during digestion. The interaction between those phenomena should be further investigated by researchers and health authorities.

Our findings show that there is a great range of values for the proposed variables presented in this study (pMeHg, MLF and RAF), but does not address the inter-population variability regarding BW and CR, nor the potential heterogeneity of MeHg distribution within fish muscle [61]. Many of these variables (BW, CR and [THg]) vary geographically and culturally, which makes the assessment of exposure to MeHg through diet difficult. This emphasizes the importance of the probabilistic risk assessment approach, where uncertainties and inter-population variability of data are included in the assessment [28].

### 4.2. The Assessment of Methylmecury Exposure through Marine Mammals Should Be a Priority

Health Canada has stated that for MeHg exposure from food the 95th percentile dose estimate obtained from probabilistic risk assessment should produce HQ < 1 [62]. Our results indicate that the inclusion of some of the proposed variables could modify the HQ for Indigenous consumers and particularly for those who consume seal liver and beluga meat (Table S3). Better assessing the values of pMeHg, MLF and RAF for foods rich in MeHg would allow for better estimates of risk and more precise consumption advisories. Presently local health services in many Canadian provinces and Territories (such as the Nunavik Regional Board of Health and Social Services (www.nrbhss.ca; accessed on 22 January 2021)) encourage the consumption of country food as a good source of nutrients but recommend that pregnant women and women of childbearing age limit their consumption of beluga meat.

Assessing exposure to MeHg through country food consumption is complex as it depends on personal consumption patterns, seasonal food availability [63] and variability of MeHg content in different tissues of country foods [2,58,61].

We must underline that a calculated HQ > 1 not necessarily leads to imminent health risks. Health Canada in its calculations of TDIs, included an uncertainty factor (UF) to account for inter-individual variability and to derive safe guidelines [64]. The TDI for the general population is based on Hg blood levels with no observable harmful effect from MeHg poisoning cases in Japan (1950–1960) and Iraq (1970), to which a UF of 10 was added [64]. For the sensitive population, the TDI comes from the data of three epidemiological studies conducted in the Seychelles and Faroes Island and New Zealand which assessed the effect of exposure to MeHg in utero on fetal neurodevelopment [64]. In this context, Health Canada used the MeHg level in maternal hair with no observable harmful effect, which was further converted into blood levels. A UF of 5 was added in order to produce the TDI [64]. Thus, HQ slightly higher to 1 will very unlikely lead to health risks. However, how far HQ can be exceeded without risks? At the moment, science does not allow an answer to this question.

Our study focused on MeHg exposure. However, balancing risks and benefits regarding country food consumption is essential and is not a trivial task since many benefits have to be considered. First, food scarcity, anemia and obesity are significant health issues for some communities in Canada, and country food brings many key nutrients that are needed to improve health. Lemire et al. [2] found that most of the commonly consumed country food in Nunavik (Canada) are poor in MeHg and rich in selenium (Se), with the exception of beluga meat and seal liver.

Further, country food consumption has also social dimensions (ex. socio-cultural and spiritual) that are usually assessed qualitatively, while health risks are measured quantitatively (ex. contaminant concentration) [65]. An overall assessment of risks and benefits requires the involvement of many disciplines such as nutrition, toxicology, sociology and public health practice [66]. Furthermore, risks and benefits can vary as a function of multiple factors, such as animal size, species, origin (aquaculture or wild), contamination, seasonal changes in consumption rates (g/day), consumer profile (age and gender) and more [7].

## 5. Conclusions

Overall, our study demonstrated the sensitivity of the MeHg exposure assessment and underlined the need to deepen our knowledge of the additional, suggested variables in order to avoid an underestimation or overestimation of the risk. Of particular importance is the need to better investigate bioaccessibility before RAF is included in a new risk assessment model.

We showed that pMeHg and RAF decreased MeHg exposure, while MLF increased it. Salmon consumption produced HQ < 1 in all cases, while the consumption of Albacore canned tuna resulted in HQ > 1 for the Canadian sensitive population, mostly when high-rate consumption is assessed. Methylmercury exposure through seal liver consumption should be investigated, since our modelling shows a great difference between the current and the alternative model. Furthermore, even though beluga *nikku* is more contaminated than beluga meat, its lesser bioaccessibility could buffer this difference and lead to similar MeHg exposure. However, validation of in vitro studies should be conducted before proposing changes in consumption guidelines.

Finally, benefits are to be considered before setting recommendations.

## Figures and Tables

**Table 1:** Values used in the probabilistic risk assessment.

	Input Variables	Point Estimate		Probability Distribution		Units	References
		CTE ^a^ (SD)	RMaE ^b^	Distribution Type	Parameters min;max		
**CR**	**Scenario 1—Fish flesh**						
	Medium consumer	22				g/day	[15]
	High consumer	40				g/day	[15]
	**Scenario 2—Marine mammals**						
	Beluga meat (pre-drying)	3.4				g/day	[2]
	Beluga *nikku*	2.3				g/day	[2]
	Seal liver	1.0				g/day	[2]
**BW**	**Scenario 1—Fish flesh**						
	General population	76.5 (15.8)	53.3	lognormal	[40;120] ***	kg	[34]
	Sensitive population	69.8 (16.3)	46.5	lognormal	[35;115] ***	kg	[34]
	**Scenario 2—Marine mammals**						
	Indigenous population	68.4 (20) *	43.9	lognormal	[38.2–129.4]	kg	[2]
**[THg]**	**Scenario 1—Fish flesh**						
	Salmon	0.03 (0.03) *	0.07	lognormal	[0–0.12]	ug/g (ww)	[15]
	Albacore canned tuna	0.35 (0.1) **	0.5	lognormal	[0.2–0.6]	ug/g (ww)	[15]
	**Scenario 2—Marine mammals**						
	Beluga meat (pre-drying)	2.0 (0.5) †	3.0	lognormal	[0.8–4.0]	ug/g (ww)	[35]
	Beluga *nikku*	5.0 (1.2)	7.2	lognormal	[2,3,4,5,6,7,8,9,10] ***	ug/g (ww)	[35]
	Ringed seal liver	19 (11.1)	23.7	lognormal	[10,11,12,13,14,15,16,17,18,19,20,21,22,23,24,25] ***	ug/g (ww)	[35]
**pMeHg**	**Scenario 1—Fish flesh**						
	Health authorities	100				%	[15]
	Literature mid data	90				%	[30]
	**Scenario 2—Marine mammals**						
	Beluga meat	65				%	[2]
	Ringed seal liver	11				%	[2]
**RAF**	**Scenario 1—Fish flesh**						
	Health authorities	100				%	[15]
	Cooked—In vitro bioaccessibility	40				%	[24,25,37,38]
	**Scenario 2—Marine mammals**						
	Beluga meat	51				%	[35]
	Beluga *nikku*	33				%	[35,39]
	Seal liver	27				%	[35,39,40]
**MLF**	**Scenario 1—Fish flesh**						
	Salmon—all cooking methods	1.35				unitless	[41,42,43,44]
	Albacore canned tuna	1				unitless	
	**Scenario 2—Marine mammals**						
	Beluga meat (raw)	1				unitless	[2]
	Beluga *nikku* (Air-dried)	2.5				unitless	[2]
	Ringed seal liver (raw)	1				unitless	[2]

^a^ Central tendency exposure (CTE) expressed as arithmetic mean; ^b^ Reasonable maximum exposure expressed as the 95th percentile of CTE; * arbitrary SD (no data available); ** weighted average; *** arbitrarily truncated to remain realistic; † [THg] for beluga meat was estimated using the [THg] of beluga *nikku* divided by MLF; CR: consumption rate; BW: body weight; [THg]: THg concentration; pMeHg: proportion of THg that is methylated; RAF: relative absorption factor; MLF: mass loss factor.

**Table 2:** Independent impact of adding a single variable in the equation of the exposure of MeHg. Results are expressed as median of MeHg daily exposure dose (μg × kg^−1^ bw) and the percentage of the distribution of exposure that exceeds the TDI value is in parentheses (no number signifies 0%). Alternative model shows the resulting MeHg daily exposure when all the proposed variables are integrated simultaneously into the exposure equation. See Table S1 for the details on variables used for each scenario. * are used to indicate a significative difference (*p* < 0.05) between the exposure distribution compared to the current model. pMeHg: proportion of THg that is methylated; RAF: relative absorption factor; MLF: mass loss factor.

**Scenario 1—Fish Flesh**	**Albacore Canned Tuna**				
**Medium Consumption Rate**					
	current model	pMeHg	RAF		alternative model
**Canadian general population**	0.100	0.090 *	0.040 *		0.036 *
**Sensitive Canadian population**	0.110 (4%)	0.010 (1%) *	0.044 *		0.040 *
**Scenario 1—Fish flesh**	**Albacore Canned Tuna**				
**High consumption rate**					
	current model	pMeHg	RAF		alternative model
**Canadian general population**	0.182 (0.1%)	0.164 *	0.073 *		0.065 *
**Sensitive Canadian population**	0.200 (50%)	0.180 (38%) *	0.08 (0.2 %) *		0.072 (0.1 %) *
**Scenario 2—Marine mammals**	**Seal liver**				
	current model	pMeHg	RAF		alternative model
**Indigenous population**	0.242 (2.7%)	0.027 *	0.065 *		0.007 *
**Scenario 2—Marine mammals**	**Beluga meat**				
	current model	pMeHg	RAF		alternative model
**Indigenous population**	0.100	0.065 *	0.051 *		0.033 *
**Scenario 2—Marine mammals**			**Beluga *nikku***		
	current model	pMeHg	RAF	MLF	alternative model
**Indigenous population**	0.068	0.044 *	0.056 *	0.169 (0.1%) *	0.036 *

## Data Availability

Data sharing not applicable. No new data were created or analyzed in this study. Data sharing is not applicable to this article.

## Supplementary Materials

The following are available online at https://www.mdpi.com/1660-4601/18/5/2565/s1, Table S1: Details of the variables used in the modelling of each scenario, Table S2: Stability of the simulations obtained by varying the number of iterations. High consumption rate of canned tuna scenario for the general population was used, Table S3: Resulting median HQ using the current and the alternative model for the general and the sensitive population (see Figure 1 and Figure 2). The percentage of the distribution of HQ > 1 is in parentheses (no number signifies 0%).
