# Supplementary material for: Effects of Speciation, Cooking and Changes in Bioaccessibility on Methylmercury Exposure Assessment for Contrasting Diets of Fish and Marine Mammals
Source: Int J Environ Res Public Health. 2021 Mar 4;18(5):2565. doi: 10.3390/ijerph18052565 (PMC7967378; doi:10.3390/ijerph18052565)
Supplement: Supplementary file 1 [file ijerph-18-02565-s001.pdf]

Supporting information for:

Effects of speciation, cooking and changes in bioaccessibility on methylmercury exposure assessment for contrasting diets of fish and marine mammals

Tania Charette [a], Gregory Kaminski [b], Maikel Rosabal [c], Marc Amyot [a]*

[a] Groupe de Recherche Interuniversitaire en Limnologie (GRIL), Université de Montréal, Département de sciences biologiques, Complexe des sciences, C.P. 6128, succ. Centre-Ville, Montréal (Québec), Canada, H3C 3J7

[b] Health Canada, 269 Laurier West, Ottawa, K1A 0K9, Canada

[c] Groupe de Recherche Interuniversitaire en Limnologie et en Environnement Aquatique (GRIL), Département des Sciences Biologiques, Université du Québec à Montréal (UQAM), 141 Avenue du Président-Kennedy, Montreal, H2X 1Y4, Canada

*Corresponding author at: Université de Montréal, Département de sciences biologiques, Complexe des sciences, C.P. 6128, succ. Centre-Ville, Montréal (Québec), Canada, H3C 3J7

E-mail address: m.amyot@umontreal.ca (M. Amyot).

Table S1. Details of the variables used in the modelling of each scenario.

**SCENARIO 1 – FISH FLESH**<br>**MEDIUM CONSUMPTION RATE**	**Salmon**	
	current model	*alternative model*
**Canadian general population**	CR-medium consumption<br>[THg] salmon<br>General population BW	CR-medium consumption<br>[THg] salmon<br>General population BW<br>+pMeHg fish flesh literature med data; +RAF cooked fish flesh; +MLF fish flesh
**Canadian sensitive population**	CR-medium consumption<br>[THg] salmon<br>Sensitive population BW	CR-medium consumption<br>[THg] salmon<br>Sensitive population BW<br>+pMeHg fish flesh literature med data; +RAF cooked fish flesh; +MLF fish flesh
**SCENARIO 1 – FISH FLESH**<br>**HIGH CONSUMPTION RATE**	**Salmon**	
	current model	*alternative model*
**Canadian general population**	CR-high consumption<br>[THg] salmon<br>General population BW	CR-high consumption<br>[THg] salmon<br>General population BW<br>+pMeHg fish flesh literature med data; +RAF cooked fish flesh; +MLF fish flesh
**Canadian sensitive population**	CR-high consumption<br>[THg] salmon<br>Sensitive population BW	CR-high consumption<br>[THg] salmon<br>Sensitive population BW<br>+pMeHg fish flesh literature med data; +RAF cooked fish flesh; +MLF fish flesh
**SCENARIO 1 – FISH FLESH**<br>**MEDIUM CONSUMPTION RATE**	**Albacore canned tuna**	
	current model	*alternative model*
**Canadian general population**	CR-medium consumption<br>[THg] tuna<br>General population BW	CR-medium consumption<br>[THg] tuna<br>General population BW<br>+pMeHg fish flesh literature med data; +RAF cooked fish flesh
**Canadian sensitive population**	CR-medium consumption<br>[THg] tuna<br>Sensitive population BW	CR-medium consumption<br>[THg] tuna<br>Sensitive population BW<br>+pMeHg fish flesh literature med data; +RAF cooked fish flesh
**SCENARIO 1 – FISH FLESH**<br>**HIGH CONSUMPTION RATE**	**Albacore canned tuna**	
	current model	*alternative model*
**Canadian general population**	CR-high consumption<br>[THg] tuna<br>General population BW	CR-high consumption<br>[THg] tuna<br>General population BW<br>+pMeHg fish flesh literature med data; +RAF cooked fish flesh
**Sensittive general population**	CR-high consumption<br>[THg] tuna<br>Sensitive population BW	CR-high consumption<br>[THg] tuna<br>Sensitive population BW<br>+pMeHg fish flesh literature med data; +RAF cooked fish flesh
**SCENARIO 2 – MARINE MAMMALS**	**Seal liver**	
	current model	*alternative model*
**Indigenous population**	CR seal liver<br>[THg] seal liver<br>Indigenous population BW	CR seal liver<br>[THg] seal liver<br>Indigenous population BW<br>+pMeHg seal liver; +RAF seal liver
**Sensitive indigenous population**	CR seal liver<br>[THg] seal liver<br>Indigenous population BW	CR seal liver<br>[THg] seal liver<br>Indigenous population BW<br>+pMeHg seal liver; +RAF seal liver
**SCENARIO 2 – MARINE MAMMALS**	**Beluga meat**	
	current model	*alternative model*
**Indigenous population**	CR beluga meat<br>[THg] beluga meat	CR beluga meat<br>[THg] beluga meat

	Indigenous population BW	Indigenous population BW<br>+pMeHg beluga meat; +RAF beluga meat
**Sensitive indigenous population**	CR beluga meat<br>[THg] beluga meat<br>Indigenous population BW	CR beluga meat<br>[THg] beluga meat<br>Indigenous population BW<br>+pMeHg beluga meat; +RAF beluga meat
**SCENARIO 2 – MARINE MAMMALS**	**Beluga nikku**	
**Indigenous population**	*current model*<br>CR beluga *nikku*<br>[THg] beluga meat<br>Indigenous population BW	*alternative model*<br>CR beluga *nikku*<br>[THg] beluga meat<br>Indigenous population BW<br>+pMeHg beluga meat; +RAF beluga *nikku*; +MLF beluga meat
**Sensitive indigenous population**	CR beluga *nikku*<br>[THg] beluga meat<br>Indigenous population BW	CR beluga *nikku*<br>[THg] beluga meat<br>Indigenous population BW<br>+pMeHg beluga meat; +RAF beluga *nikku*; +MLF beluga meat

Table S2. Stability of the simulations obtained by varying the number of iterations. High consumption rate of canned tuna scenario for the general population was used.

Iteration number	Median TDI ($\mu g \times kg^{-1}$ bw)	% of at-risk population*
10,000	0.182	0.05
50,000	0.182	0.056
100,000	0.182	0.059
1,000,000	0.181	0.0572
10,000,000	0.181	0.05772

*when TDI obtained is superior to TDI threshold of 0.47 $\mu g \times kg^{-1}$ bw.

Table S3. Resulting median HQ using the current and the alternative model for the general and the sensitive population (see Figs. 1 and 2). The percentage of the distribution of HQ > 1 is in parentheses (no number signifies 0%).

**SCENARIO 1 – FISH FLESH**<br>**MEDIUM CONSUMPTION RATE**	**Salmon**		**Canned tuna**	
	current model	*alternative model**	*current model*	*alternative model**
**Canadian general population**	0.01	0.006	0.2	0.08
**Sensitive Canadian population**	0.03	0.02	0.5 (4%)	0.2
**SCENARIO 1 – FISH FLESH**<br>**HIGH CONSUMPTION RATE**	**Salmon**		**Canned tuna**	
	current model	*alternative model**	*current model*	*alternative model**
**Canadian general population**	0.02	0.01	0.4 (0.1%)	0.1
**Sensitive Canadian population**	0.06	0.03	1.0 (50%)	0.4 (0.1%)
**SCENARIO 2 – MARINE MAMMALS**	**Seal liver**			
	current model	*alternative model**		
**Indigenous population**	0.5 (2.7%)	0.06		
**Sensitive Indigenous population**	1.2 (69%)	0.1		
**SCENARIO 2 – MARINE MAMMALS**	**Beluga meat**		**Beluga *nikku***	
	current model	*alternative model**	*current model*	*alternative model**
**Indigenous population**	0.2	0.1	0.1	0.08
**Sensitive Indigenous population**	0.5 (1.9%)	0.2	0.3 (0.01%)	0.2
